# Correlation analysis between biomechanical characteristics of lower extremities during front roundhouse kick in Taekwondo and effective scores of electronic protectors

**DOI:** 10.3389/fbioe.2024.1364095

**Published:** 2024-06-21

**Authors:** Mengyao Jia, Yong Ma, Ruifeng Huang, Lin Liu, Zhaoyi Wang, Shijie Lin, Qian Peng, Jun Xiong, Weitao Zheng

**Affiliations:** ^1^ Engineering Research Center of Sports Health Intelligent Equipment of Hubei Province, Wuhan Sports University, Wuhan, China; ^2^ Research Center of Sports Equipment Engineering Technology of Hubei Province, Wuhan Sports University, Wuhan, China; ^3^ Key Laboratory of Sports Engineering of General Administration of Sports of China, Wuhan Sports University, Wuhan, China; ^4^ Department of Physical Education, Intelligent Sports Engineering Research Center, Northwest Polytechnical University, Xi’an, China; ^5^ School of Competitive Sports, Wuhan Sports University, Wuhan, China

**Keywords:** Taekwondo, electronic protective gear, front roundhouse kick, biomechanics, effective scores

## Abstract

**Objective:** The purpose of this study is to analyze the inherent relationship between the score values and the biomechanical characteristics of the forward kicking motion, we aim to identify the fundamental variables influencing the score values of the forward kicking motion and establish the key biomechanical factors that effectively trigger scoring in the forward kicking motion.

**Methods:** The DaeDo electronic scoring system was used with the Vicon optical motion capture system and the Kistler 3D force platform to obtain kinematic and kinetic variables of the front roundhouse kick motion. Linear bivariate correlation analysis and principal component analysis were used to analyze the associations between kinematic, kinetic variables, and scoring values, and summarize key biomechanical factors for effectively scoring.

**Results:** The peak ankle plantar flexion angle and knee extension torque of the kicking leg showed a significant negative correlation with scoring values (*r* < 0, *p* < 0.05), while other variables showed no statistical significance. The peak knee flexion angle and hip extension angular velocity of the supporting leg showed a significant positive correlation with scoring values (*r* > 0, *p* < 0.01), while the peak ankle plantar flexion torque showed a significant negative correlation with scoring values (*r* < 0, *p* < 0.05), and other variables showed no statistically significant correlation. The absolute values of eigenvectors of the first and second principal components, which included hip angular velocity, ankle angle, knee torque, and hip torque, were relatively large, indicating their strong influence on effective scoring triggering.

**Conclusion:** Maintaining ankle dorsiflexion and a larger knee flexion angle in the kicking leg is favorable for triggering scoring. Higher knee flexion angle and hip extension angular velocity in the supporting leg are also advantageous for triggering scoring. “Body posture” and “Strength” are key factors that effectively trigger scoring.

## Introduction

Competitive Taekwondo is a combative sport characterized by direct physical contact and intense confrontation (Pieter et al., 2012). Its action traits include fast technical movements, short contact time, and frequent changes in techniques and tactics (Lin et al., 2023). In past competitions, referees had to subjectively judge the effectiveness of athletes’ strikes (Zhi et al., 2010). Because there is a blind spot in the field of vision when athletes come into contact with each other, judging results were prone to unfairness, partiality, and delays (Fan et al., 2010; Qing et al., 2014). To address these issues, significant revisions were made to the Taekwondo competition regulation after the Rio Olympics, leading the sport into the era of electronic protective gear (Lin and Gao, 2020). As the official certified brand of the World Taekwondo Federation, DaeDo electronic protective gear have been widely used in major events such as the Olympics and World Championships (Nan and Jian, 2017).

In competitive Taekwondo, the effectiveness of strikes is not only influenced by strength but also closely related to the strike precision, angle, and hit rate on the target area (Razman and Chong, 2019). The features of competitive Taekwondo techniques are powerful strike to the opponent’s trunk or head with the lower limbs (Geßlein et al., 2020). Therefore, athletes need to focus on enhancing the explosiveness of muscles and the coordination of movements during their training to improve strike power and speed. Additionally, by designing appropriate technical actions, conducting professional physical training, and enhancing body flexibility in postural control, the strength and accuracy of strikes can be further improved.

Taekwondo have high extremely requirements for flexibility and specialized strength from athletes (Bridge et al., 2014). It involves frequent use of kicking and striking techniques in order to eliminate opponents through scoring. The scoring areas of electronic protective gear mainly include the opponent’s trunk and head. The characteristic of front roundhouse kick involves significant limb movements in a short amount of time, requiring athletes to possess high levels of flexibility and coordination. Additionally, athletes need to have excellent specialized strength, including muscle strength, explosive power, and postural control (Nan et al., 2017). Through training and techniques accumulation, athletes can swiftly use front roundhouse kick actions for both offensive and defensive purposes, making it difficult for opponents to predict and respond. Moreover, the feature of this action minimizes the vulnerabilities exposed by athletes, effectively enhancing their defensive capabilities (Son et al., 2020).

The current literature on the front roundhouse kick mainly involves technical and tactical adjustments under the new rules (He and Pang, 2019), biomechanical characteristics of specific techniques (Aloui et al., 2022; Liu et al., 2023), and the impact of training on kicking techniques (Ojeda-et al., 2021; Sant’ana et al., 2021). However, in studies related to the use of DaeDo electronic body protectors to trigger scoring in Taekwondo, there is a lack of research on key biomechanical factors related to the scoring values of the front roundhouse kick. In other sports, research on key biomechanical factors has been able to determine the primary factors that determine competition results (Bullock et al., 2009; Crossland et al., 2011; Tomasevicz et al., 2020). It is hypothesized that there is relatively little attention given to the relationship between the value of effective scoring and the kinematic and kinetic characteristics of the kicking motion when using electronic protectors to trigger valid scoring.

Therefore, this study aims to investigate the correlation between the kicking speed, posture control in movements (Liu et al., 2023), and the scoring values by electronic protective gear of the front roundhouse kick, using high-speed camera systems and a three-dimensional force platform. By analyzing the intrinsic relationship between scoring values and the biomechanical characteristics of the front roundhouse kick, this study aims to reveal the underlying variables that influence scoring values and summary key biomechanical factors for effectively triggering score with the front roundhouse kick. This will provide Taekwondo coaches and athletes with variables to assess the effectiveness of the front roundhouse kick in triggering the electronic protective gear, thus offering theoretical support for improving the training effectiveness of the front roundhouse kick technique in Taekwondo and triggering scores effectively during competitions.

The novelty of this study lies in the investigation of the correlation between the biomechanical characteristics of the lower extremities during the front roundhouse kick in Taekwondo and the effective scores of electronic protectors. This study aims to analyze the intrinsic relationship between scoring values and biomechanical factors, providing insights into the variables that influence scoring values and identifying key biomechanical factors for effectively triggering scores with the front roundhouse kick. This research fills the gap in the existing literature by focusing on the biomechanical factors related to scoring values in Taekwondo using electronic protective gear.

## Materials and methods

### Study population

In this study, we used G Power 3.1.9.2 to calculate the required sample size with an effect size of 0.7. Under the conditions of α = 0.05 and a statistical power of 95%, 13 participants were needed. Based on the weight class set for men’s Taekwondo competitions in the Olympic Games, Finally, 15 excellent athletes from Wuhan Sports University Taekwondo sports training team, (with a weight range of 54–87 kg), were recruited as the participants for this experiment (18.00 ± 2.20 years, 182.15 ± 8.62 cm, 70.00 ± 14.82 kg). All participants had experience participating in regional-level or higher-level competitions and were in a healthy physical condition that would not influence their kicking performance. None of the participants engaged in intense exercise within 24 h prior to the experiment, and they had no significant injuries or abnormalities in their anatomical structure or function within the past 6 months. All participants exhibited good overall physical condition and athletic abilities.

### Event division

**FIGURE 1:**
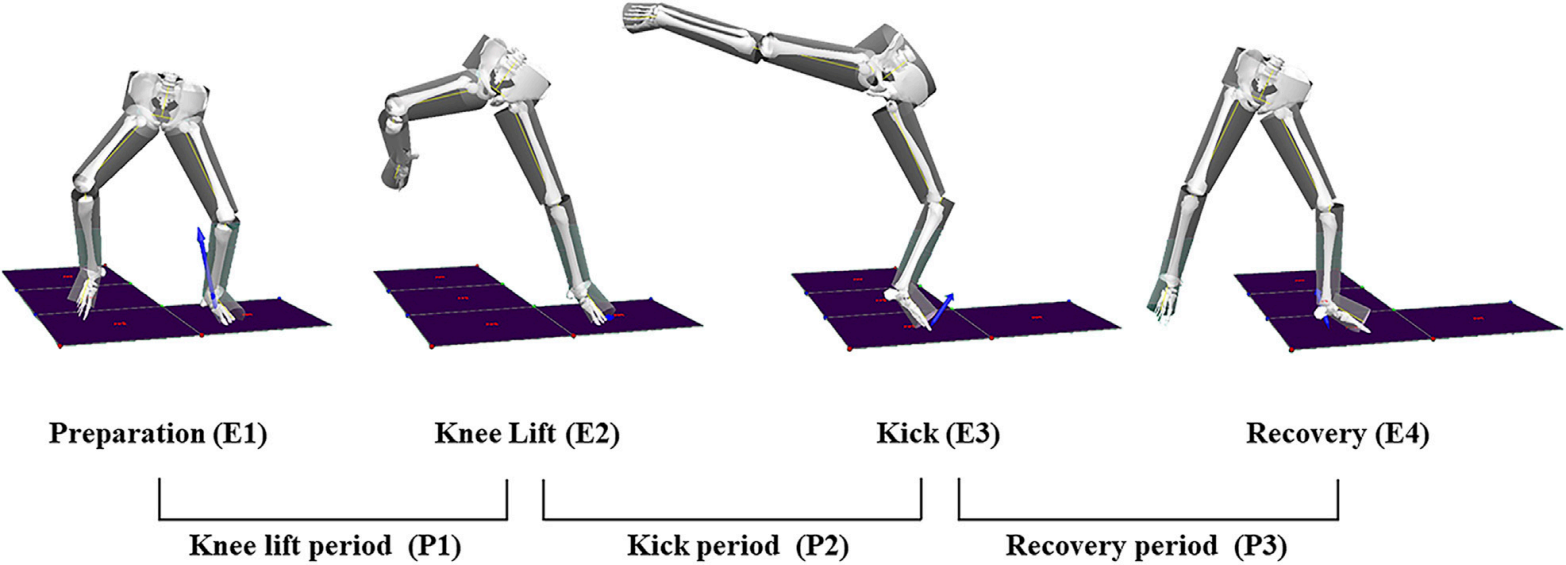
Time and period division of front roundhouse kick.

Based on the characteristics of the front roundhouse kick motion, relevant studies (Jeong et al., 2021; Jian et al., 2020; Qiu et al., 2021), and interviews with coaches and athletes, this study divided the front roundhouse kick movement into four events and two phases, as shown in Figure 1.

Preparation Moment (E1): The moment when the striking leg leaves the ground and the moment when the attacking foot rise from the force platform with no ground reaction force. Lift Knee Moment (E2): The moment of minimal flexion of the striking leg knee joint and the moment of maximum angle of the striking leg knee joint. Kick Moment (E3): The moment of maximum extension of the striking leg knee joint and the moment of minimum angle of the striking leg knee joint. Recovery Moment (E4): The moment the striking leg lands and the moment the attacking foot touches the force platform with ground reaction force emerge. Lift Knee Phase (P1): From the preparation moment to the lift knee moment, E1-E2. Striking Phase (P2): From the lift knee moment to the Kick moment, E2-E3. Recovery Phase (P3): From the kick moment to the recovery moment, E3-E4. In this study, the striking leg is defined as the lower limb of one side that hit the dummy, and the lower limb in contact with the ground is defined as the supporting leg.

### Measurements and procedures

The experiment utilized 9 infrared high-speed cameras (T40, Vicon, United Kingdom) with a sampling frequency of 200 Hz and Marker balls with a diameter of 14 mm to collect kinematic data. The Kistler 3D force platform (9260AA6, Switzerland) with 4 independent force plates of size 60 × 40 cm and a sampling frequency of 1,500 Hz was used to collect dynamic data. Synchronization between the Vicon system and the Kistler system was achieved through a digital signal converter. The scoring values were recorded using the Korean DaeDo electronic protective gear and scoring system (specifically designed for the Olympic Games), which included torso protectors, electronic helmets, and electronic foot guards. During the experiment, the dummy was in an upright position, and the electronic protective gear was worn by the dummy. The electronic protective gear was wirelessly connected to the computer through a signal receiver.

**FIGURE 2:**
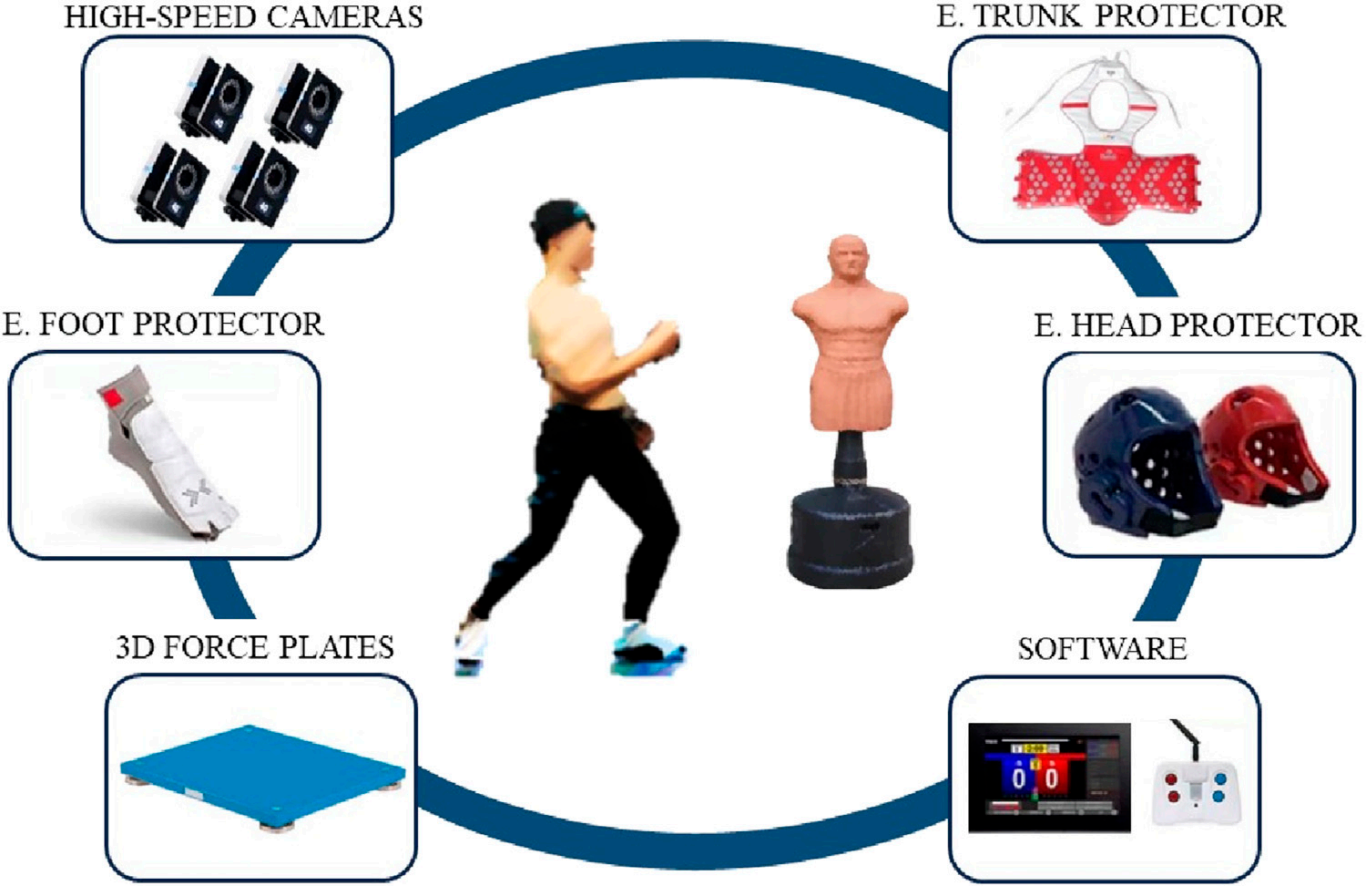
Test schematic diagram.

This study has been reviewed and approved by the ethics review of Wuhan Sports University (NO. 2022048) and participants were instructed on the testing procedures prior to the test. The test procedures are illustrated in Figure 2. Preparations before the test involved initializing and calibrating the Vicon 3D motion capture system, preheating and calibrating the Kistler 3D force platform, as well as testing and calibrating the Daedo electronic protective gear. Synchronization of data between the Vicon and Kistler systems was confirmed. Participants underwent a health assessment and provided informed consent form, being informed about the experimental procedures and precautions. Participants engaged in a 15-min warm-up activity, followed by attaching reflective Marker balls. Once the attachment was completed, participants were instructed to rapidly strike the dummy equipped with the electronic protective gear according to the predefined global coordinate direction and the position of the target dummy. The height of the dummy was adjusted to match the habitual striking height of the participants. After the athletes fully adapted, the experimenters checked the signals of the electronic protective gear and set the electronic scoring system to the corresponding weight class of the participant’s match. Data collection commenced thereafter.

In taekwondo competitions, in order to complete movements with higher quality and achieve the goal of scoring, the collected actions in this study aimed to simulate realistic combat situations as closely as possible. The participants performed a combination of step-in and front roundhouse kick, as shown in Figure 1. After each movement of the subject is completed, the same coach determined whether the test action met the technical standards for a front roundhouse kick, and five successful actions were selected as test samples. The dominant kicking limb of all subjects was the right leg.

### Variables selection and coordinate system, joint angle definition

Definition of Joint Coordinate System and Joint Angles according to the setup in Visual 3D. The joint coordinate system is defined with three axes in reference to the participant’s body. The axes include the coronal axis (*X*-axis, flexion/extension), the sagittal axis (*Y*-axis, abduction/adduction), and the vertical axis (*Z*-axis, internal/external rotation). The following joint angles are defined: Ankle Joint Angle: The angle between the foot and the line extending from the leg. Knee Joint Angle: The angle between the lower leg and the line extending from the thigh. Hip Joint Angle: The angle between the thigh and the line extending from the trunk.

During the front roundhouse kick, the joint movement sequence of the striking leg involves pelvic rotation, followed by simultaneous flexion of the knee joint, hip abduction/adduction, and trunk rotation until contact with the target is made (Chun et al., 2020). Studies on front roundhouse kick in taekwondo typically analyze the movement process using variables such as maximum joint angles, peak joint angular velocities, peak joint moments, and vertical ground reaction forces (Moreira and Paula, 2017; Sant’ana et al., 2017; Chang et al., 2021; Jeong et al., 2021; Liu et al., 2021; Pei and Jian, 2021; Zhao and Jia, 2022; Zhi et al., 2022). The selected variables for this study are as follows:

1) Range of Motion of Hip, Knee, and Ankle Joints: The maximum flexion/extension angles (°) of the hip, knee, and ankle joints for both the striking leg and the supporting leg. Flexion of the hip, knee, and dorsiflexion of the ankle are defined as positive directions.
2) Velocity of Motion of Hip, Knee, and Ankle Joints: The peak angular velocities (rad/s) of the hip, knee, and ankle joints for both the striking leg and the supporting leg are measured. The direction of joint angular velocities follows the same definition as the joint angles.
3) Kinematics: The peak knee joint moment (N·m), ankle joint moment (N·m), hip joint moment (N·m) for both the kicking leg and the supporting leg are measured. Additionally, the vertical ground reaction force (vGRF), after being normalized to body weight (BW), is also measured.
4) Score Value: The score value (Unitless) corresponding to the effective front roundhouse kick action is measured.

### Data processing

Select the three optimal movements from each participant’s data, and export them as C3D files after marker labeling and gap filling in Vicon software. Then import the C3D files into Visual 3D software. The lower limb model is used for static modeling, and relevant variables are computed. The kinematic and kinetic data are filtered with cut-off frequencies of 10 Hz and 25 Hz, respectively (Ibrahim et al., 2014; Moreira and Paula, 2017; Yi et al., 2018). The kinetic data are normalized to each participant’s body weight multiplied by their height. The kinematic, kinetic, and score values of the three selected data sets are averaged.

### Statistical analysis

In this study, statistical analysis of the data was performed using SPSS 19.0 software. Linear bivariate correlation analysis was conducted to examine the relationships between kinematics, kinetics, muscle activity, and score values (Winter et al., 2001; Keylock et al., 2022). The significance level was set at *p* < 0.05 to determine statistical significance. One asterisks (*) denote *p* < 0.05, while double asterisks (**) denote *p* < 0.01.

**FIGURE 3:**
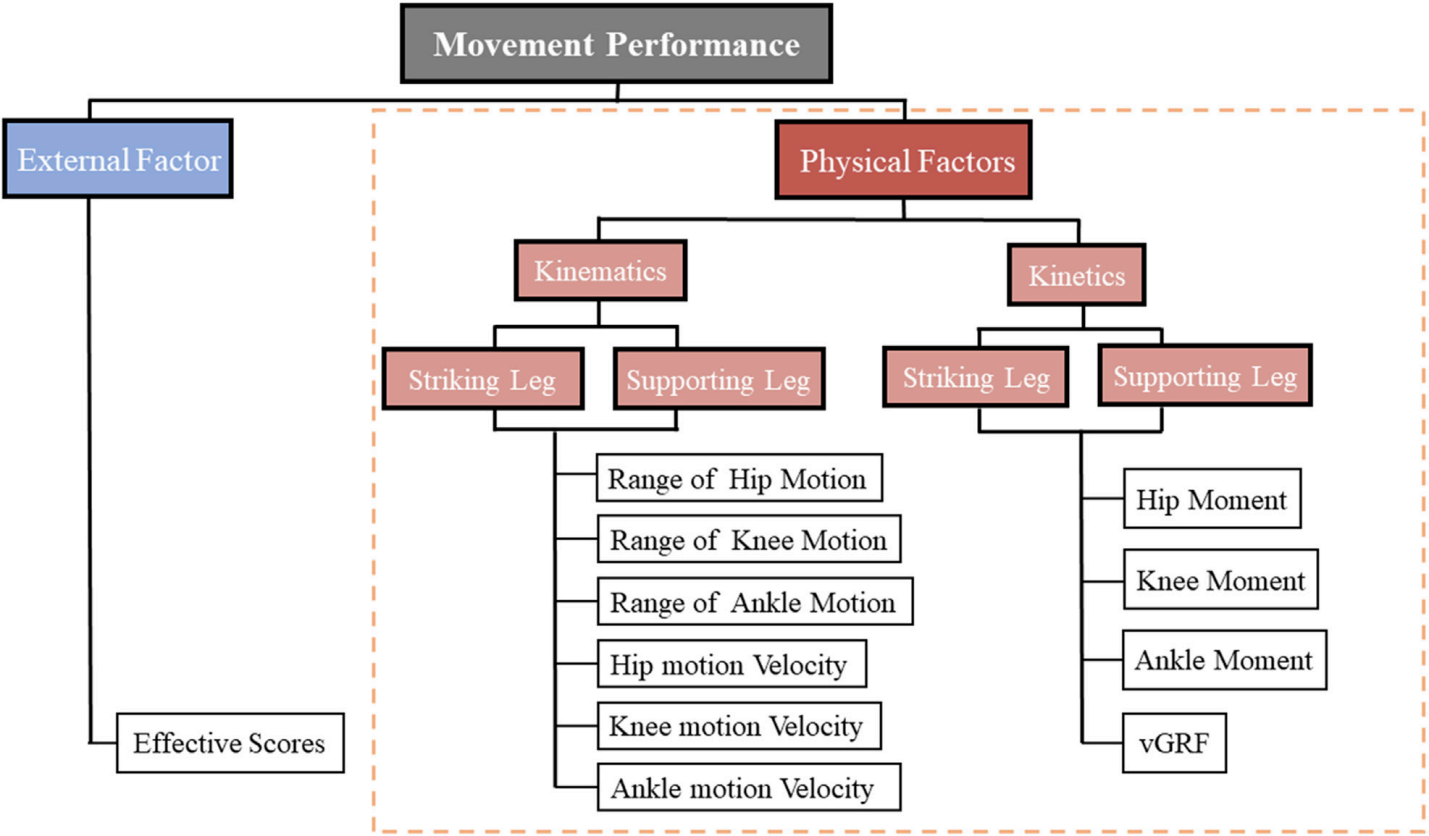
10 variables that may lead to a successful or unsuccessful trigger score.

Based on the research variables, we chose ten key variables that may influence the success or failure of athletes in triggering scores, as shown in Figure 3. These variables include hip, knee, ankle joint angles, angular velocities, joint moments, and vertical ground reaction forces. All variables were computed using Visual 3D software. Principal Component Analysis (PCA) was performed using the “factoextra” package in R software (Warmenhoven et al., 2021; Jiang et al., 2023). The sample correlation matrix was calculated based on the sample data matrix, and the eigenvalues and eigenvectors of the correlation matrix were obtained. The principal components and their contributions were determined based on these eigenvalues and eigenvectors. Finally, key variables to trigger scores in the front roundhouse kick movements were identified through PCA.

## Results

### Kinematic and kinetic characteristics of the striking leg and supporting leg of front roundhouse kick

**FIGURE 4:**
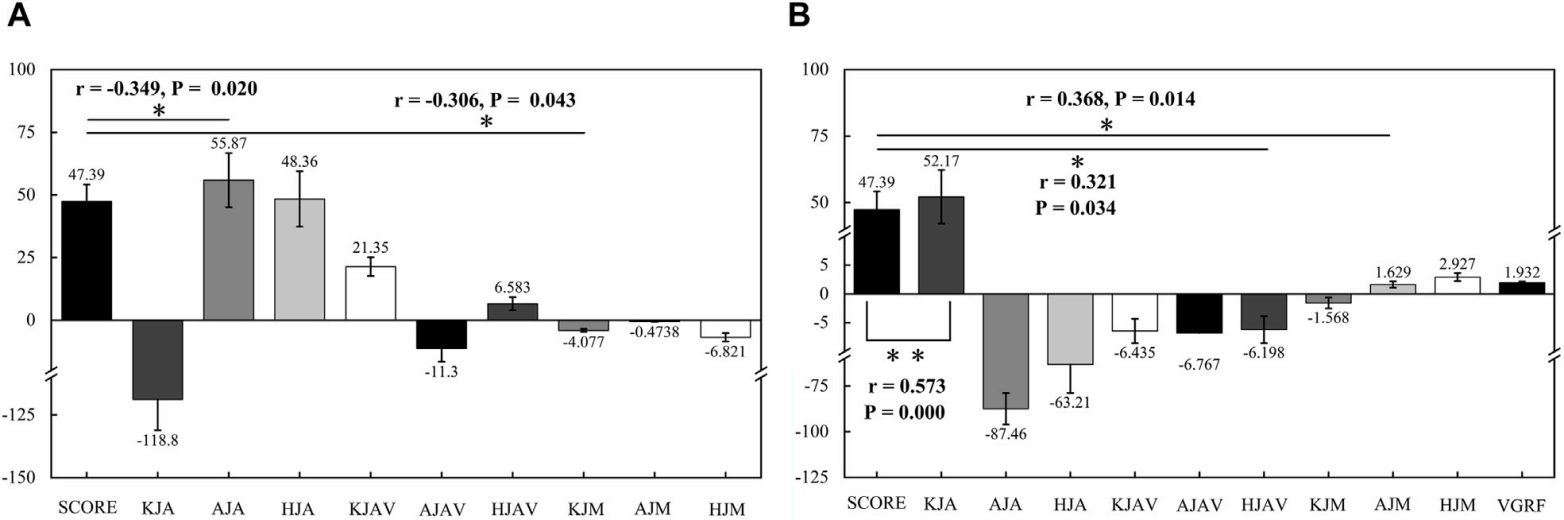
**(A)** kinematics and kinetic characteristics of the striking leg; **(B)** kinematics and kinetic characteristics of the supporting leg (*) significant discrepancy *p* < 0.05 (**) very significant discrepancy, *p* < 0.01).

As shown in Figure 4A, for striking leg, significant negative correlations were observed between the peak dorsiflexion angle of the ankle joint and the peak extension moment of the knee joint (*r* = −0.349, *p* = 0.020; *r* = −0.306, *p* = 0.043), as well as no statistically significant relationships between the peak flexion angle of the knee joint, peak extension angle of the hip joint, peak extension angular velocity of the knee joint, peak plantar flexion angular velocity of the ankle joint, peak extension angular velocity of the hip joint, peak plantar flexion moment of the ankle joint, peak extension moment of the hip joint and the score values (*p* > 0.05).

Furthermore, as shown in Figure 4B, for supporting leg, there were significant positive correlations between the peak flexion angle of the knee joint, peak extension angular velocity of the hip joint, and peak dorsiflexion moment of the ankle joint with the score values (*r* = 0.573, *p* = 0.000; *r* = 0.321, *p* = 0.034; *r* = 0.368, *p* = 0.014). Similarly, no statistically significant relationships were found between the peak dorsiflexion angle of the ankle joint, peak extension angle of the hip joint, peak extension angular velocity of the knee joint, peak dorsiflexion angular velocity of the ankle joint, peak extension moment of the knee joint, peak flexion moment of the hip joint, peak vertical ground reaction force and the score values (*p* > 0.05).

### Principal component analysis was conducted on the lower limb kinematic and kinetic variables

An assessment was conducted on the suitability of the research data for principal component analysis. From Table 1, it can be observed that the Kaiser-Meyer-Olkin (KMO) measure is 0.608, surpassing the threshold of 0.6, thereby satisfying the prerequisite requirement for conducting principal component analysis. This indicates that the data can be utilized for principal component analysis. Furthermore, the data successfully passed the Bartlett’s sphericity test (*p* < 0.05), affirming the appropriateness of employing principal component analysis on the research data.

**TABLE 1:** KMO and Bartlett’s test.

Test method	Evaluation index	Results
Kaiser-Meyer-Olkin (KMO) Test		0.608
Bartlett’s Sphericity Test	*p*	0.000

Table 2 presents an analysis of the principal component extraction and the amount of information extracted. From the table, it is evident that a total of 6 principal components were extracted in the principal component analysis, with eigenvalues all greater than 1. The variance explained by these 6 principal components are 29.560%, 13.605%, 10.518%, 9.725%, 7.802%, and 5.394% respectively, with a cumulative variance explained of 76.603%. Furthermore, the analysis yielded 6 principal components, with their corresponding weighted variance explained as follows: 29.560/76.603 = 38.59%; 13.605/76.603 = 17.76%; 10.518/76.603 = 13.73%; 9.725/76.603 = 12.70%; 7.802/76.603 = 10.18%; 5.394/76.603 = 7.04%.

**TABLE 2:** Variance explained by principal components.

Number	Eigenvalue			Principal component extraction		
	Eigenvalue	Variance explained %	Cumulative %	Eigenvalue	Variance explained %	Cumulative %
1	5.616	29.560	29.560	5.616	29.560	29.560
2	2.585	13.605	43.165	2.585	13.605	43.165
3	1.998	10.518	53.683	1.998	10.518	53.683
4	1.848	9.725	63.407	1.848	9.725	63.407
5	1.482	7.802	71.209	1.482	7.802	71.209
6	1.025	5.394	76.603	1.025	5.394	76.603
7	0.814	4.285	80.888	—	—	—
8	0.694	3.652	84.540	—	—	—
9	0.570	2.999	87.539	—	—	—
10	0.558	2.937	90.477	—	—	—
11	0.412	2.167	92.644	—	—	—
12	0.340	1.792	94.435	—	—	—
13	0.299	1.576	96.011	—	—	—
14	0.248	1.304	97.316	—	—	—
15	0.182	0.960	98.275	—	—	—
16	0.118	0.620	98.895	—	—	—
17	0.094	0.493	99.388	—	—	—
18	0.070	0.369	99.757	—	—	—
19	0.046	0.243	100.000	—	—	—

Table 3 illustrates the information extraction of the principal components for the research items and their corresponding relationships. From the table, it can be observed that all research items have communalities values higher than 0.4, indicating a strong correlation between the research items and the principal components, enabling effective information extraction. Therefore, this study extracted relevant information from the loading coefficient table of each principal component. PC1, PC2, PC5, and PC6 primarily contain joint angles and angular velocities of the offensive leg, joint torques, angular velocities, and vertical ground reaction force of the support leg. These indicators reflect the posture of the offensive leg and the muscle recruitment ability of the support leg. Hence, PC1 is defined as the body posture and muscular strength factor. PC3 mainly encompasses joint angles and angular velocities of both the offensive and support legs, thus referred to as the body posture factor. PC4 primarily involves joint torques of the offensive leg and is defined as the muscular strength factor. The 6 principal components mainly reflect the body posture and muscular strength of athletes performing sideways kicking movements.

**TABLE 3:** Loadings matrix of principal components.

Indicators	Loading coefficient						Common variance
	PC1	PC2	PC3	PC4	PC5	PC6	
Peak Knee Joint Angle (OFF)	0.683	0.457	−0.253	0.026	−0.073	0.322	0.849
Peak Ankle Joint Angle (OFF)	−0.565	−0.237	−0.478	−0.337	−0.013	0.450	0.921
Peak Hip Joint Angle (OFF)	−0.676	−0.435	0.010	0.169	0.279	−0.201	0.793
Peak Knee Joint Angular Velocity (OFF)	−0.276	0.274	0.688	0.172	0.040	0.273	0.730
Peak Ankle Joint Angular Velocity (OFF)	0.711	−0.056	−0.039	−0.005	−0.515	−0.031	0.777
Peak Hip Joint Angular Velocity (OFF)	−0.505	−0.291	0.147	−0.255	0.373	0.019	0.566
Peak Knee Joint Moment (OFF)	0.133	−0.627	−0.074	0.551	−0.055	0.424	0.902
Peak Ankle Joint Moment (OFF)	0.260	0.178	0.378	0.702	0.130	−0.052	0.754
Peak Hip Joint Moment (OFF)	0.414	−0.331	−0.030	0.569	0.287	−0.124	0.703
Peak Knee Joint Angle (SUP)	−0.290	−0.179	−0.618	0.443	−0.137	−0.204	0.754
Peak Ankle Joint Angle (SUP)	0.587	0.102	0.108	0.022	0.381	0.458	0.722
Peak Hip Joint Angle (SUP)	0.741	0.285	−0.164	0.139	0.020	0.066	0.681
Peak Knee Joint Angular Velocity (SUP)	0.378	−0.337	0.590	−0.312	−0.223	−0.041	0.754
Peak Ankle Joint Angular Velocity (SUP)	0.721	−0.401	0.167	−0.093	−0.347	−0.198	0.877
Peak Hip Joint Angular Velocity (SUP)	0.754	−0.082	−0.371	−0.089	0.032	−0.111	0.735
Peak Knee Joint Moment (SUP)	0.633	0.251	0.034	−0.286	0.475	−0.215	0.819
Peak Ankle Joint Moment (SUP)	−0.214	0.807	−0.202	0.130	0.195	−0.184	0.826
Peak Hip Joint Moment (SUP)	−0.593	0.313	0.278	0.218	−0.370	−0.057	0.714
vGRF (SUP)	−0.523	0.465	−0.077	0.117	−0.394	0.111	0.677

Note: When the absolute value of the loading coefficient is greater than 0.4, it indicates a strong correlation between the research item and the principal component. The principal component can effectively extract information, where ‘−’ represents a negative correlation. OFF, offensive leg; SUP, support leg.

## Discussion

The research findings indicate a strong correlation between the peak joint angles and peak joint angular velocities of the selected offensive leg and support leg with the effective triggering of scores. It is speculated that this is because the movement between joints in the front roundhouse kick action proceeds in a proximal-to-distal sequence, where the peak velocities in the proximal segments are smaller than those in the distal segments. Joints accelerate sequentially from the proximal segments to the distal segments, enabling the foot to reach maximum linear velocity (Estevan et al., 2015). In the accelerating phase of the striking leg, If the knee joint moment remains high, it hampers the transmission of speed from the proximal to the distal segments, thus failing to accelerate the foot to the conditions for effective triggering of scoring. On the other hand, a larger knee extension moment may be disadvantageous for knee flexion. According to Quinzi et al. (2013), a lower knee flexion angle leads to a smaller rotation area of the striking leg, and Ying et al. (2011) also indicated that insufficient knee flexion significantly affects the rotational speed of the striking leg with the hip joint as the center, thereby affecting the transmission of inertia torque to the striking phase (Crossland et al., 2011). Similarly, Liu et al. (2021) suggests that the amplitude of knee flexion directly affects the speed of the striking phase. A greater internal/external rotation angular velocity of the knee joint during the knee lifting phase is more conducive to increasing the knee flexion and extension velocity, and the mass distribution of the lower extremity is closer to the axis of rotation. According to the results of principal component analysis, peak hip joint moment and peak knee joint moment are the key variables affecting effective triggering score of the roundhouse kick action. Hoelbling et al. (2020) have already shown that for the front roundhouse kick, the hip flexors must counteract the hip extension moment, which is caused by the knee joint’s inertial torque in the direction of extension during the angular acceleration process. When the knee joint extends, the angular velocity of the hip joint is relatively lower, producing a greater hip flexion moment, which increases the foot’s linear velocity during the transmission to the distal segment, thus more conducive to triggering scoring.

The present study found that the peak joint torque values of the offensive leg and support leg are also crucial factors in triggering effective score values. One study (Machado et al., 2010) suggests that the striking techniques requires contact between the back of the foot and the electronic protective gear worn by the dummy, and the contact area between the foot and the electronic protective gear directly affects whether the final score can be triggered. During the kick moment, the inversion/eversion of the ankle joint affects the vertical component of contact between the foot and the opponent, leading to a decrease in the contact area between the foot and the opponent, thus affecting the score. The results of this study indicate that larger dorsiflexion angle at the ankle joint adversely affects trigger scoring. This is because during the kick moment, the contact between the dorsum of the foot and the electronic protective gear is not completely perpendicular, the angles and moments at the ankle joint generate components in the sagittal and vertical axes that are not sufficient to trigger scoring. Therefore, in matches where electronic protective gear is used, apart from factors like speed and strength, the angle of the ankle joint is also a crucial factor influencing scoring. Athletes should pay attention to reducing inversion/eversion of the ankle joint in their daily training.

We also found a significant positive correlation between the hip joint extension angular velocity of the supporting leg, the knee joint flexion angle and the scoring value. There was also a significant negative correlation between the ankle joint plantar flexion moment and the scoring value. In order to trigger scoring, the goal of striking is to generate sufficient speed at the distal end of the striking leg, requiring close coordination between the lower limbs and the trunk (Ervilha et al., 2020). During the knee lifting phase, the trunk undergoes significant rotation around its axis to obtain a larger moment of inertia, thus driving the lower limbs to complete the striking action (Shi et al., 2012). For the supporting leg, its stability is a key factor in ensuring trigger scores (Giroux et al., 2016). Excessive knee joint flexion/extension moment hinders the transfer of moment of inertia from the trunk to the striking leg, lowering the quality of subsequent actions. Previous studies have shown a significant correlation between the extension moment generated at the hip joint and the flexion angular velocity of knee joint (Fong and Tsang, 2012). The sagittal axis ground reaction force can provide moment for hip joint rotation, accelerating body rotation to drive the striking leg to complete the striking action. At the same time, the forward kinetic energy of the body is converted into elastic potential energy stored in the lower limb muscles, which is then released during take-off, generating a larger vertical ground reaction force (Yu and Ji, 2020). This feedbacks into the knee lifting phase, increasing the height of knee-lifting for athletes. The striking leg generates significant impact force at the moment of striking, but the supporting leg prevents the body from moving backward after the impact, thus making attack of the subsequent kicking more powerful (Flanagan and Salem, 2008).

## Conclusion and prospect

The peak ankle dorsiflexion angle, peak knee extension moment of the kicking leg, peak knee flexion angle of the supporting leg, peak hip extension angular velocity, and peak ankle dorsiflexion moment were identified as key biomechanical indicators influencing effective scoring in the forward kicking motion. There was a strong correlation between the joint angle peaks, joint angular velocity peaks, joint moment peaks, and vertical ground reaction force of the supporting leg with effective scoring. The factors of “body posture” and “muscle strength” were identified as crucial factors for executing the forward kicking motion and triggering effective scoring.

In summary, the key factors for effectively triggering scoring in electronic protective gear are body posture and strength. Taekwondo coaches and athletes can focus on developing specialized training sessions for body posture and strength training, thereby improving effective scoring ratios in competitive competitions. Due to the limitations of this study, the definition of the principal component “muscle strength” factor was characterized solely by joint torque. Future research needs to confirm the effect of lower limb joint muscle strength on effectively triggering scores. Furthermore, this study only examined the overall correlation between peak values of relevant variables and score values. Future research should explicitly identify the key biomechanical factors for effectively triggering scores at different moments (preparation, knee-lift, striking, recovery).

## Data Availability

The raw data supporting the conclusion of this article will be made available by the authors, without undue reservation.

## References

[B1] Aloui A. Tayech A. Mejri M. A. Makhlouf I. Clark C. C. Granacher U. (2022). Reliability and validity of a new taekwondo-specific change-of-direction speed test with striking techniques in elite taekwondo athletes: a pilot study. Front. Physiology 13, 774546. 10.3389/fphys.2022.774546 PMC9086405 35557973

[B2] Bridge C. A. Ferreira Da Silva Santos J. Chaabene H. Pieter W. Franchini E. (2014). Physical and physiological profiles of taekwondo athletes. Sports Med. 44, 713–733. 10.1007/s40279-014-0159-9 24549477

[B3] Bullock N. Gulbin J. P. Martin D. T. Ross A. Holland T. Marino F. (2009). Talent identification and deliberate programming in skeleton: ice novice to Winter Olympian in 14 months. J. Sports Sci. 27 (4), 397–404. 10.1080/02640410802549751 19191166

[B4] Chang W. G. Lin K. Y. Chu M. Y. Chow T. H. (2021). Differences in pivot leg kinematics and electromyography activation in various round house kicking heights. J. Sports Sci. Med. 20 (3), 457–465. 10.52082/jssm.2021.457 34267585 PMC8256524

[B5] Chun J. X. Wei J. L. DE H. L. Hai L. Y. (2020). Effect of hitting speed of four tactical movements of taekwondo back roundhouse kick and its enlightenment for sports training. J. Chengdu Sport Univ. 46 (1), 114–120. 10.15942/j.jcsu.2020.01.018

[B6] Crossland B. W. Hartman J. E. Kilgore J. L. Hartman M. J. Kaus J. M. (2011). Upper-body anthropometric and strength measures and their relationship to start time in elite luge athletes. J. Strength & Cond. Res. 25 (10), 2639–2644. 10.1519/JSC.0b013e318207ed7a 21873904

[B7] Ervilha U. F. Fernandes F. D. M. Souza C. C. D. Hamill J. (2020). Reaction time and muscle activation patterns in elite and novice athletes performing a taekwondo kick. Sports Biomech. 19 (5), 665–677. 10.1080/14763141.2018.1515244 30274543

[B8] Estevan I. Falco C. Silvernail J. F. Jandacka D. (2015). Comparison of lower limb segments kinematics in a Taekwondo kick. An approach to the proximal to distal motion. J. Hum. Kinet. 47 (1), 41–49. 10.1515/hukin-2015-0060 26557189 PMC4633266

[B9] Fan L. Zhi H. G. Liang Z. L. (2010). First time application of electronic taekwondo protective gear in China. J. Wuhan Inst. Phys. Educ. 44 (10), 73–77+96. 10.3969/j.issn.1000-520X.2010.10.016

[B10] Flanagan S. P. Salem G. J. (2008). Lower extremity joint kinetic responses to external resistance variations. J. Appl. Biomechanics 24 (1), 58–68. 10.1123/jab.24.1.58 18309184

[B11] Fong S. S. Tsang W. W. (2012). Relationship between the duration of taekwondo training and lower limb muscle strength in adolescents. Hong Kong Physiother. J. 30 (1), 25–28. 10.1016/j.hkpj.2011.11.004

[B12] GeßLEIN M. Rüther J. Bail H. J. Schuster P. Krutsch W. Wolpert A. K. (2020). Injury incidence rates and profiles in elite taekwondo during competition and training. Int. J. Sports Med. 41 (1), 54–58. 10.1055/a-1021-1776 31747701

[B13] Giroux C. Rabita G. Chollet D. Guilhem G. (2016). Optimal balance between force and velocity differs among world-class athletes. J. Appl. Biomechanics 32 (1), 59–68. 10.1123/jab.2015-0070 26398964

[B14] He Y. Pang J. P. (2019). Development trend of men’s taekwondo techniques and tactics under new rules: taking world champion lee dae-hoon as an example. J. Wuhan Sports Univ. 53 (12), 82–87. 10.15930/j.cnki.wtxb.2019.12.012

[B15] Hoelbling D. Baca A. Dabnichki P. (2020). Sequential action, power generation and balance characteristics of a martial arts kick combination. Int. J. Perform. Analysis Sport 20 (5), 766–781. 10.1080/24748668.2020.1774730

[B16] Ibrahim Haridy A. M. Ibrahim Atta I. (2014). Analytical study of some biomechanical variables and their effect on achievement level in the Pole vault. J. Appl. Sports Sci. 4 (1), 32–39. 10.55384/2790-4237.1043

[B17] Jeong H. S. O'Sullivan D. M. Jeong D. H. Lee S. Y. (2021). Sports injuries and illnesses after implementation of the web-based surveillance system in world Taekwondo. J. Athl. Train. 56 (11), 1232–1238. 10.4085/330-19 33657209 PMC8582623

[B18] Jian J. S. Ying H. Z. Yu L. Zhi H. W. Chao H. F. Yuan L. (2020). The covariate analysis of kinematic characteristics of taekwondo athletes’ ankle joints in turning kick with different fatigues. Chin. J. Sports Med. 39 (12), 924–931. 10.3969/j.issn.1000-6710.2020.12.002

[B19] Jiang X. Xu D. Fang Y. Bíró I. Baker J. S. Gu Y. (2023). PCA of running biomechanics after 5 km between novice and experienced runners. Bioengineering 10 (7), 876. 10.3390/bioengineering10070876 37508903 PMC10376576

[B20] Keylock L. Felton P. Alway P. Brooke-Wavell K. Peirce N. King M. (2022). Lumbar bone mineral adaptation: the effect of fast bowling technique in adolescent cricketers. Med. Sci. Sports Exerc. 54 (3), 438–446. 10.1249/mss.0000000000002820 34711706

[B21] Lin D. S. Gao Z. H. (2020). The characteristics of the use of turning kick skills and training countermeasures in taekwondo competition under new rules. J. Beijing Sport Univ. 43 (10), 114–123. 10.19582/j.cnki.11-3785/g8.2020.10.011

[B22] Lin L. Yong M. Shi J. L. Qian P. Ling L. D. Jun X. (2023). Biomechanics research on laterality effect between dominant and non-dominant during front cross kick in taekwondo. J. Wuhan Sports Univ. 57 (1), 73–81. 10.3969/j.issn.1000-520X.2023.01.010

[B23] Liu T. T. Lin Y. C. Tang W. T. Hamill J. Chang J. S. (2021). Lower-limb kinematic characteristics of Taekwondo kicks at different attack angles. Int. J. Perform. Analysis Sport 21 (4), 519–531. 10.1080/24748668.2021.1924526

[B24] Liu L. Jia M. Y. Ma Y. Lin S. J. Peng Q. Xiong J. (2023). Biomechanics research on laterality effect between dominant and non-dominant during double roundhouse kick in the competitive taekwondo. Heliyon 9 (10), e20843. 10.1016/j.heliyon.2023.e20843 37876451 PMC10590780

[B25] Machado S. M. Osório R. A. L. Silva N. S. Magini M. (2010). Biomechanical analysis of the muscular power of martial arts athletes. Med. Biol. Eng. Ccomputing 48, 573–577. 10.1007/s11517-010-0608-z 20390460

[B26] Moreira P. Paula L. (2017). Kinesiologic description of the round house kick: a brief review. J. Athl. Enhanc. 6 (1), 1–6. 10.4172/2324-9080.1000250

[B27] Nan Z. Jian M. G. (2017). Technical and tactical characteristics of taekwondo men's 58kg champion zhao shuai in 2016 Rio Olympics. J. Beijing Sport Univ. 40 (2), 95–99. 10.19582/j.cnki.11-3785/g8.2017.02.015

[B28] Nan Z. Wei J. L. Jian B. Z. Hai L. Y. (2017). The competition pattern and development trend of taekwondo in the last three Olympic Games. J. Shandong Sport Univ. 33 (4), 92–96. 10.14104/j.cnki.1006-2076.2017.04.017

[B29] Ojeda-Aravena A. Herrera-Valenzuela T. Valdés-Badilla P. Cancino-López J. Zapata-Bastias J. García-García J. M. (2021). Inter-Individual variability of a high-intensity interval training with specific techniques vs. repeated sprints program in sport-related fitness of taekwondo athletes. Front. Physiology 12, 766153. 10.3389/fphys.2021.766153 PMC8637814 34867471

[B30] Pei F. C. Jian J. S. (2021). Kinematic feature analysis of the risk of acute injury of the lateral collateral ligament caused by whip leg. J. Xi'an Phys. Educ. Univ. 38 (2), 219–225. 10.16063/j.cnki.issn1001-747x.2021.02.014

[B31] Pieter W. Fife G. P. O'Sullivan D. M. (2012). Competition injuries in taekwondo: a literature review and suggestions for prevention and surveillance. Br. J. Sports Med. 46 (7), 485–491. 10.1136/bjsports-2012-091011 22661697

[B32] Qing C. Z. Zhi H. G. Shan L. Yu H. C. (2014). Scoring characters of referees in world qualifying trials for 2012 London Olympics. China Sport Sci. Technol. 50 (3), 65–68. 10.3969/j.issn.1002-9826.2014.03.011

[B33] Qiu S. Z. Ben Y. F. Yu Y. Z. (2021). On the correlations among scoring factors in world competitive trampoline. J. Xi'an Phys. Educ. Univ. 38 (3), 379–384. 10.16063/j.cnki.issn1001-747x.2021.03.018

[B34] Quinzi F. Camomilla V. Felici F. DI Mario A. Sbriccoli P. (2013). Differences in neuromuscular control between impact and no impact roundhouse kick in athletes of different skill levels. J. Electromyogr. Kinesiol. 23 (1), 140–150. 10.1016/j.jelekin.2012.09.006 23089236

[B35] Razman R. Chong R. W. L. (2019). Reliability and validity of a taekwondo electronic body protector. J. Sports Eng. Technol. 233 (2), 202–209. 10.1177/1754337118815

[B36] Sant'Ana J. Franchini E. Da Silva V. Diefenthaeler F. (2017). Effect of fatigue on reaction time, response time, performance time, and kick impact in taekwondo roundhouse kick. Sports Biomech. 16 (2), 201–209. 10.1080/14763141.2016.1217347 27592682

[B37] Sant'Ana J. Sakugawa R. L. Diefenthaeler F. (2021). The effect of a pace training session on internal load and neuromuscular parameters in taekwondo athletes. Front. Physiology 12, 710627. 10.3389/fphys.2021.710627 PMC8370830 34413790

[B38] Shi M. L. Yi F. B. Yu P. Q. (2012). Biomechanical analysis on principles of lower limb whiplash movement. China Sport Sci. Technol. 48 (4), 101–107+136. 10.3969/j.issn.1002-9826.2012.04.015

[B39] Son B. Cho Y. J. Jeong H. S. Lee S. Y. (2020). Injuries in Korean elite taekwondo athletes: a prospective study. Int. J. Environ. Res. Public Health 17 (14), 5143. 10.3390/ijerph17145143 32708739 PMC7399793

[B40] Tomasevicz C. L. Ransone J. W. Bach C. W. (2020). Predicting bobsled pushing ability from various combine testing events. J. Strength & Cond. Res. 34 (9), 2618–2626. 10.1519/JSC.0000000000002489 29533357

[B41] Warmenhoven J. Bargary N. Liebl D. Harrison A. Robinson M. A. Gunning E. (2021). PCA of waveforms and functional PCA: a primer for biomechanics. J. Biomechanics 116, 110106. 10.1016/j.jbiomech.2020.110106 33429072

[B42] Winter E. M. Eston R. G. Lamb K. L. (2001). Statistical analyses in the physiology of exercise and kinanthropometry. J. Sports Sci. 19 (10), 761–775. 10.1080/026404101317015429 11561673

[B43] Yi J. Zhi L. Dian K. C. (2018). Influence of different sole thickness on biomechanical parameters of human lower extremity. J. Xi'an Phys. Educ. Univ. 35 (6), 731–741. 10.16063/j.cnki.issn1001-747x.2018.06.015

[B44] Ying H. Hai J. D. Zhong Q. J. (2011). Biomechanics research on the offensive back turning kick in long distance of elite tae kwon do players. J. Beijing Sport Univ. 34 (4), 64–67. 10.19582/j.cnki.11-3785/g8.2011.04.017

[B45] Yu L. Ji H. Z. (2020). Kinematics analysis on different positions' serve skills in world elite male tennis players. China Sport Sci. 40 (8), 58–64. 10.16469/j.css.202008006

[B46] Zhao L. L. Jia Y. Z. (2022). Characteristics of extensor group of lower limbs in drop jumping. J. Xi'an Phys. Educ. Univ. 39 (2), 224–231. 10.16063/j.cnki.issn1001-747x.2022.02.012

[B47] Zhi H. G. Ju T. F. Wen G. R. Zhi M. Q. (2010). The effects of new rule and electronic protective gear application on taekwondo technique. China Sport Sci. Technol. 46 (4), 86–89+98. 10.3969/j.issn.1002-9826.2010.04.014

[B48] Zhi Y. L. Liang C. Song K. Y. Xu B. F. Yu Z. L. Ye H. W. (2022). Effect of short approach run on steps on kinematics and muscle activity of takeoff leg of step in triple jump. J. Xi'an Phys. Educ. Univ. 38 (4), 484–492. 10.16063/j.cnki.issn1001-747x.2021.04.013

